# Leadless biventricular left bundle and endocardial lateral wall pacing versus left bundle only pacing in left bundle branch block patients

**DOI:** 10.3389/fphys.2022.1049214

**Published:** 2022-12-14

**Authors:** Marina Strocchi, Nadeev Wijesuriya, Mark K. Elliott, Karli Gillette, Aurel Neic, Vishal Mehta, Edward J. Vigmond, Gernot Plank, Christopher A. Rinaldi, Steven A. Niederer

**Affiliations:** ^1^ School of Biomedical Engineering and Imaging Sciences, King’s College London, London, United Kingdom; ^2^ Guy’s and St Thomas’ NHS Foundation Trust, London, United Kingdom; ^3^ BioTechMed-Graz, Graz, Austria; ^4^ Gottfried Schatz Research Center, Medical University of Graz, Graz, Austria; ^5^ NumeriCor GmbH, Graz, Austria; ^6^ University of Bordeaux, CNRS, Bordeaux, France; ^7^ IHU Liryc, Bordeaux, France

**Keywords:** cardiac resynchronization therapy, left bundle branch block, leadless pacing, dyssynchrony, conduction system pacing, left bundle pacing, endocardial pacing

## Abstract

Biventricular endocardial (BIV-endo) pacing and left bundle pacing (LBP) are novel delivery methods for cardiac resynchronization therapy (CRT). Both pacing methods can be delivered through leadless pacing, to avoid risks associated with endocardial or transvenous leads. We used computational modelling to quantify synchrony induced by BIV-endo pacing and LBP through a leadless pacing system, and to investigate how the right-left ventricle (RV-LV) delay, RV lead location and type of left bundle capture affect response. We simulated ventricular activation on twenty-four four-chamber heart meshes inclusive of His-Purkinje networks with left bundle branch block (LBBB). Leadless biventricular (BIV) pacing was simulated by adding an RV apical stimulus and an LV lateral wall stimulus (BIV-endo lateral) or targeting the left bundle (BIV-LBP), with an RV-LV delay set to 5 ms. To test effect of prolonged RV-LV delays and RV pacing location, the RV-LV delay was increased to 35 ms and/or the RV stimulus was moved to the RV septum. BIV-endo lateral pacing was less sensitive to increased RV-LV delays, while RV septal pacing worsened response compared to RV apical pacing, especially for long RV-LV delays. To investigate how left bundle capture affects response, we computed 90% BIV activation times (BIVAT-90) during BIV-LBP with selective and non-selective capture, and left bundle branch area pacing (LBBAP), simulated by pacing 1 cm below the left bundle. Non-selective LBP was comparable to selective LBP. LBBAP was worse than selective LBP (BIVAT-90: 54.2 ± 5.7 ms vs. 62.7 ± 6.5, *p* < 0.01), but it still significantly reduced activation times from baseline. Finally, we compared leadless LBP with RV pacing against optimal LBP delivery through a standard lead system by simulating BIV-LBP and selective LBP alone with and without optimized atrioventricular delay (AVD). Although LBP alone with optimized AVD was better than BIV-LBP, when AVD optimization was not possible BIV-LBP outperformed LBP alone, because the RV pacing stimulus shortened RV activation (BIVAT-90: 54.2 ± 5.7 ms vs. 66.9 ± 5.1 ms, *p* < 0.01). BIV-endo lateral pacing or LBP delivered through a leadless system could potentially become an alternative to standard CRT. RV-LV delay, RV lead location and type of left bundle capture affect leadless pacing efficacy and should be considered in future trial designs.

## Introduction

Cardiac resynchronization therapy (CRT) is an effective treatment for heart failure patients with left bundle branch block (LBBB). Conventional CRT is delivered through a right ventricular (RV) lead, normally implanted in the RV apex, and a transvenous left ventricular (LV) lead implanted in the coronary sinus targeting the latest activated region, to achieve biventricular (BIV) pacing. Despite a large amount of evidence of CRT benefits on patients with LV dyssynchrony, between 30% and 50% of patients receiving CRT do not experience target clinical improvements (Sieniewicz et al., 2019). CRT inefficacy has been attributed to many factors, including challenging coronary sinus anatomy, presence of scar and phrenic nerve stimulation (Butter et al., 2021). Furthermore, transvenous leads are associated with risk of lead infection or rupture, sometimes requiring risky extraction procedures (Bernard, 2016).

Endocardial pacing and conduction system pacing (CSP) have emerged as potential alternatives to standard CRT, to reduce the rate of non-responders. Biventricular endocardial (BIV-endo) pacing delivered through an RV apical lead and an LV endocardial lead was shown to be more beneficial than standard CRT (Behar et al., 2016). BIV-endo pacing is not restricted by the coronary sinus anatomy, provides faster access to the ventricular fast conducting system and preserves physiological transmural activation from endocardium to epicardium (Prinzen et al., 2009; Hyde et al., 2015). However, the implantation of an LV endocardial lead requires lifelong anticoagulation to reduce the risk of stroke (Morgan et al., 2016). Furthermore, ventricular resynchronization relies on the fusion of two unphysiological wavefronts spreading from the RV apex and the LV free wall. CSP has the potential to restore the native synchronous activation of the patient prior to the block. CSP delivered through His bundle pacing (HBP) was shown to be more beneficial than standard CRT (Arnold et al., 2018), but it requires high pacing thresholds and is challenging to perform, restricting this method to centers with experienced operators. Compared to HBP, left bundle pacing (LBP) offers lower and more stable thresholds with a larger area to target, making it easier to perform. Response to LBP might however depend on atrioventricular (AV) delay optimization (Strocchi et al., 2020b; Lin et al., 2020) and type of left bundle capture (selective vs. non-selective vs. septal myocardium pacing). Often, LBP is delivered through a lead screwed deep in the septum from the RV side, although pacing is not always achievable through this method. Pacing through the LV septum may be more reliable, but this would increase the risk of stroke with a conventional lead pacing system.

As mentioned above, BIV-endo pacing and LV LBP applicability is hindered by the risk of stoke following lead implantation. These risks can be attenuated by delivering pacing through a leadless pacing system. The WiSE-CRT system (EBR Systems Inc., Sunnyvale, CA) is the only commercially available leadless LV pacing system (Auricchio et al., 2014). The system consists of a battery connected to an ultrasound transducer implanted subcutaneously between the ribs and an LV leadless endocardial receiver electrode. It also requires a device capable of performing continuous RV pacing, such as a transvenous pacemaker, implantable cardioverter defibrillator (ICD), or a leadless RV pacemaker such as MICRA™ (Medtronic, Minneapolis, NN). The transmitter and the battery then detect the RV pacing spike and, within 10 ms, the ultrasound transmitter emits several ultrasound pulses to locate the receiving LV electrode, normally located in the lateral wall (Auricchio et al., 2014; Reddy et al., 2017; Sieniewicz et al., 2020). Once the electrode is located, a longer pulse is emitted and converted by the electrode to a pacing stimulus, resulting in BIV-endo lateral wall pacing. LBP delivery could also be improved by delivering LV septal pacing through an LV leadless system. The feasibility, safety and short-term response of LBP through the WiSE-CRT system was assessed by Elliott et al. in patients and pigs (Elliott et al., 2021; Elliott et al., 2022). In (Elliott et al., 2022), LBP alone was performed first with a temporary mapping catheter to ensure correct targeting of the left bundle. Once left bundle capture was achieved, the leadless electrode was implanted and anchored to perform leadless LBP. Short-term safety and response were assessed, although there remain questions about long-term effects of this implantation technique. Despite the development of BIV-endo pacing, LBP and leadless pacing (Behar et al., 2016; Reddy et al., 2017; Sieniewicz et al., 2020; Elliott et al., 2021), there are still questions about how the RV lead location (apex vs. septum), LV lead location (lateral wall vs. septum), RV-LV delay and LBP type of capture affect response.

This study aims to use computational electrophysiology to address unanswered clinical questions about BIV-endo pacing and LBP delivered through leadless pacing in LBBB patients. We run simulations to mimic the protocol used in (Elliott et al., 2022) to quantify the efficacy of leadless LBP (e.g., LBP in conjunction with RV pacing) vs. LBP alone in resynchronizing ventricular activation. The effect of RV pacing location and RV-LV delay on response is quantified by repeating the pacing protocol with an RV apical lead and an RV septal lead, and by increasing the RV-LV delay from 5 ms to 35 ms. The effect of the type of left bundle capture is assessed by simulating selective LBP, non-selective LBP and left bundle branch area pacing (LBBAP) by pacing 1 cm below the left bundle. In addition, we compare leadless LBP to BIV-endo lateral wall pacing to assess the effect of the LV electrode location on simulated electrical response.

## Methods

### Electrophysiology simulations

We performed electrophysiology simulations on twenty-four chamber heart geometries generated from heart failure patients and published as part of a previous study (Strocchi et al., 2020a). The meshes were made of linear tetrahedral elements, with an average resolution of 1 mm. Local ventricular activation times were computed using the Eikonal equation (Neic et al., 2017). The Eikonal model computes the local time t_a_(**x**) at each node with location **x** within a domain Ω, provided an initial activation time t_0_ at an initial stimulus location Γ and the conduction velocity (CV) tensor **V**, containing the squared CV along the fiber, sheet and normal to sheet directions.

$$\sqrt{\nabla t_{a}{\left(x\right)}^{T}V\nabla t_{a}\left(x\right)}=1,x\in \Omega$$

$$t_{a}\left(x\right)=t_{0},x\in \Gamma$$

In this study, the domain Ω consisted of the ventricular myocardium and the His-Purkinje network. The stimulus locations Γ were set to the first node of the His and to the CRT stimuli locations (as detailed below) during baseline and pacing simulations, respectively. Ventricular myocardium was simulated as a transversely isotropic conduction medium with fibers and cross-fibers CV set to 0.6 m/s and 0.24 m/s (Taggart et al., 2000), respectively, while the His-Purkinje CV was set to 3.0 m/s (Ono et al., 2009). The Eikonal equation was solved with the Fast Iterative Algorithm, as described in (Neic et al., 2017).

For each geometry, we generated a His-Purkinje network with proximal LBBB. The Purkinje tree was grown on the endocardial surfaces of the ventricles and accounted for five fascicles: LV anterior, LV posterior, LV septal, RV septal and RV moderator band. The location for the fascicle root points was provided according to early activation sites in the Durrer maps, using the universal ventricular coordinates (UVCs) (Bayer et al., 2018) to ensure consistency across the meshes. The ventricular myocardium and the His-Purkinje system were coupled by connecting each terminal point of the Purkinje network with the points of the myocardium within 1 mm distance to allow for stimulus propagation from the Purkinje system to the myocardium and *vice versa*. The anterograde and retrograde delay were set to 10 ms and 3 ms, respectively, based on (Behradfar et al., 2014) Further details about the His-Purkinje network generation can be found in the Supplement and in (Gillette et al., 2021; Gillette et al., 2022). In the supplement, we also provide a validation of our model during LBBB baseline by comparing the simulated activation pattern and metrics against electrocardiographic imaging data.

**FIGURE 1:**
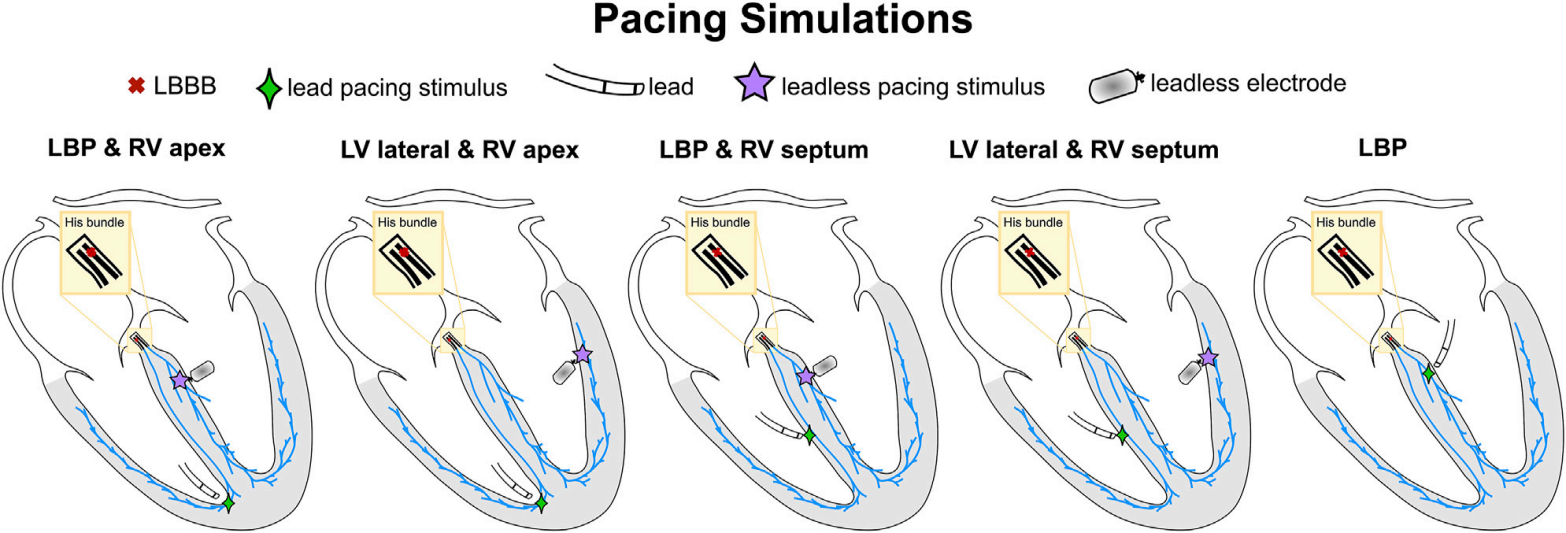
Schematic representation of pacing simulations. The red cross shows the proximal LBBB introduced along the His. The green diamonds and the purple stars represent the pacing locations through a standard lead and a leadless electrode, respectively.

Figure 1 summarizes the CRT simulations performed in this study. The pacing locations listed below provide stimuli regions Γ (see Eikonal equation above) where we prescribe an activation time. The earliest stimulus location was assigned with an activation time of 0 ms, while other pacing locations (if any) were stimulated according to a specified delay. Leadless BIV pacing was simulated by stimulating the RV at the apex and the LV at the lateral wall (BIV-endo lateral) or at the septum, selectively targeting the left bundle (BIV-LBP). Unless otherwise specified, the RV-LV delay was set to 5 ms, simulating a nearly simultaneous LV stimulus after RV pacing spike detection through the transducer, in keeping with real-world techniques. LBP through a standard lead was simulated by pacing the LBP selectively without RV pacing. LBP alone was simulated both with and without AV delay optimization. In LBP simulations with AV delay optimization, we paced the left bundle and the first node of the His to simulate two activation waves, one starting at the LBP site and one travelling from the atria down to the ventricles. We stimulated these sites with delays of 0 ms, e.g. left bundle paced when the activation wave enters the His, 10 ms, 20 ms or 30 ms, e.g. the left bundle is stimulated 30 ms after the activation wave enters the His. We also simulated LBP-ahead pacing, where the left bundle is stimulated 10 ms, 20 ms or 30 ms before the activation wave enters the His. We then selected the simulation that provided the shortest activation times, defined according to the activation metrics described below. All simulations apart from LBP with optimized AV delay were carried out under the assumption that pacing completely overwrites the patient’s native activation. All pacing stimuli were prescribed with a radius of 1.5 mm.

Patients with septal scar might have different response to pacing compared to patients with proximal LBBB alone. To investigate the effect of the presence of septal scar on our analysis, we ran simulations in the presence of septal scar, and we presented our results in the supplement. We mapped a patient-specific scar and border zone geometry from a publicly available LV mesh (Mendonca Costa et al., 2019) using the UVCs (Bayer et al., 2018). The UVCs were computed on the LV of our twenty-four meshes and on the LV of the mesh the scar was mapped from. Then, the scar and border zone were mapped by finding the closest element in UVC distance on the target mesh. Scar tissue was simulated as non-conducting, while the border zone was assigned with an isotropic CV of 0.24 m/s (Mendonca Costa et al., 2019). The Purkinje overlapping the scar was also simulated as non-conducting.

### Electrical response

We studied the effect of RV lead location and RV-LV delay on response to BIV-endo lateral pacing and BIV-LBP, and how the type of LBP capture affects BIV-LBP efficacy. To this end, BIV-endo lateral pacing and BIV-LBP simulations were repeated with an increased RV-LV delay of 10, 20, 30 and 35 ms. The RV pacing stimulus was then moved from the apex to the septum to quantify changes in response caused by the RV lead location. Finally, to investigate the effect of left bundle capture, BIV-LBP simulations were repeated with three different types of left bundle capture: selective, non-selective and septal myocardium capture (e.g., left bundle branch area pacing, LBBAP). Selective LBP was simulated by selectively pacing the left bundle. Non-selective pacing and LBBAP were simulated by extending the LBP stimulus to the surrounding myocardium and by pacing the LV septum1 cm below the left bundle, respectively.

To quantify LV and BIV synchrony, we computed LVAT-95 and BIVAT-90 as the shortest interval to activate 95% of the LV and 90% of the ventricles, respectively. Additionally, we quantified the LV and BIV dyssynchronous index (LVDI and BIVDI) as the standard deviation of the LV and BIV activation times, respectively. The area around the four cardiac valves were excluded when computing activation times.

Simulation results were compared using one-way analysis of variance (ANOVA). Post-hoc comparison analysis was performed to see which pairwise comparisons were statistically different using the Tukey’s honestly significant difference test.

## Results

### Comparison between leadless BIV pacing and selective LBP

**FIGURE 2:**
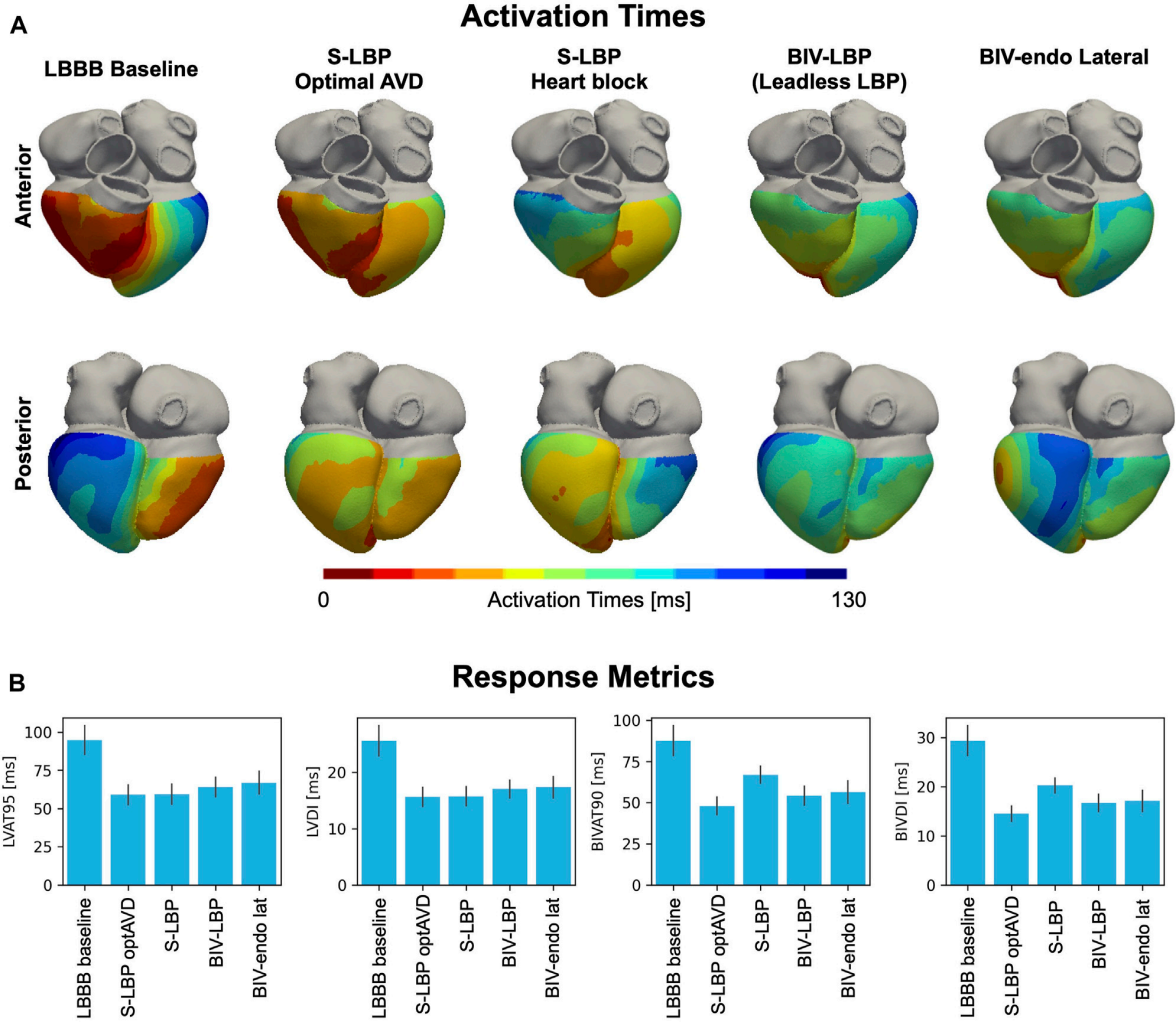
**(A)** Simulated activation times: Red and blue areas represent early and late activated regions, respectively. We show simulations during LBBB baseline, selective LBP (S-LBP) with and without AV delay optimization, leadless S-LBP (BIV-LBP, e.g., with an additional RV apical stimulus) and BIV-endo pacing with the LV lead in the lateral wall (BIV-endo lat). **(B)** Response metrics: BLVAT-95, LVDI, BIVAT-90 and BIVDI are shown for baseline and pacing. The bar plot represents the mean while the black segments represent ±standard deviation.

We used computational electrophysiology to mimic the pacing protocol in (Elliott et al., 2022), with LBP alone followed by leadless LBP (e.g., BIV-LBP). BIV-LBP was simulated with an RV apical stimulus and selective LBP with an RV-LV delay of 5 ms, while selective LBP alone was simulated by pacing the left bundle, both with and without optimized AV delay. Figure 2 shows the simulated activation times (A) and the response metrics (B) during baseline and pacing. Selective LBP with optimized AV delay led to optimal synchrony, with shorter LV activation compared to baseline, where the LV was activated later than the RV. In the presence of complete AV block (e.g. when AV delay optimization is not possible), the LV was still activated quickly, but the RV activation was delayed because the patient’s intrinsic activation was unable to travel down from the atria along the right bundle to activate the RV. The RV stimulus introduced during BIV-LBP improved RV activation compared to selective LBP with AV block. In terms of activation metrics (Figure 2B), selective LBP with and without AV delay optimization and BIV-LBP significantly shortened LV and BIV activation times compared to baseline. Selective LBP with optimized AV delay was better than BIV-LBP, although the difference was not statistically significant for BIVAT-90 (BIVAT-90: 54.2 ± 5.7 ms P = 0.09, LVAT-95: 64.0 ± 6.3 ms, *p* < 0.01). However, when AV delay optimization was not possible, BIV-LBP achieved better synchrony compared to LBP without RV pacing (BIVAT-90: 54.2 ± 5.7 ms vs. 66.9 ± 5.1 ms, *p* < 0.01). Although selective LBP with optimized AV delay delivered through a standard pacing lead remains the best LBP delivery method for patients without AV block or atrial fibrillation, when AV delay optimization is not possible a leadless system offers better synchrony than LBP alone.

We compared BIV-LBP and selective LBP alone with BIV-endo lateral wall pacing, as the LV lateral wall is the standard location for the LV leadless electrode implantation. The last column of Figure 2A shows activation times simulated during BIV-endo lateral pacing. Pacing from the LV endocardial lateral wall improved activation compared to baseline, as the LV lateral wall stimulus shortened LV activation while the RV apical stimulus kept RV activation short. Similarly, to BIV-LBP and selective LBP alone, BIV-endo lateral wall pacing significantly shortened LV and BIV activation times compared to baseline. However, BIV-endo lateral wall pacing was significantly worse than selective LBP alone with optimized AV delay in terms of both ventricular (BIVAT-90: 48.0 ± 5.3 ms vs. 56.4 ± 6.8 ms, *p* < 0.01) and LV activation times (LVAT-95: 59.0 ± 6.5 ms vs. 66.9 ± 7.4 ms, *p* < 0.01). BIV-endo lateral wall pacing was however comparable to BIV-LBP (BIVAT-90: 54.2 ± 5.7 ms vs. 56.4 ± 6.8 ms, *p* = 0.9), indicating that placing the LV leadless electrode in the LV lateral wall does not result in a significantly different response compared to selectively targeting the left bundle.

In the supplement, we analyzed the effect of septal scar on our results by repeating the comparisons above in the presence of non-conductive tissue in the septum. Our results show that septal scar makes BIV-LBP and LBP completely ineffective because the LBP stimulus does not capture the healthy myocardium or Purkinje. On the other hand, BIV-endo lateral pacing remains effective.

### The effect of prolonged RV-LV delay

**FIGURE 3:**
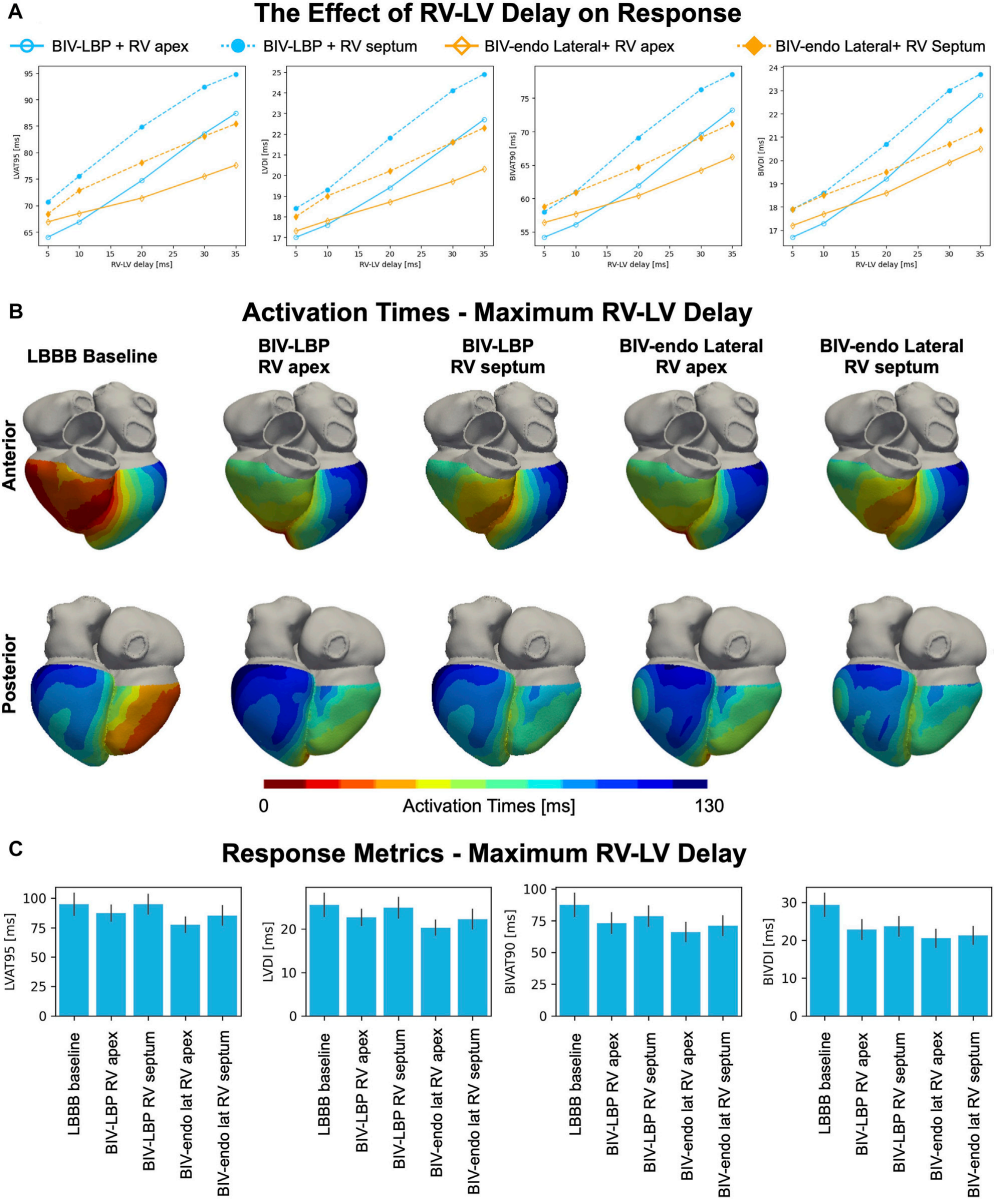
The effect of RV-LV delay and RV pacing location on response. **(A)** Mean LVAT-95, LVDI, BIVAT-90 and BIVDI achieved with BIV-endo lateral pacing or BIV-LBP with RV apical or septal pacing for different RV-LV delays. **(B)** Simulated activation times during baseline and pacing for maximum RV-LV delay (35 ms). **(C)** Response metrics (mean ± standard deviation) simulated during baseline and pacing.

Although BIV-LBP and BIV-endo lateral wall pacing were comparable with an RV-LV delay of 5 ms, response to pacing might be affected by the RV-LV delay or by the RV lead location. To test this, we repeated BIV-LBP and BIV-endo lateral wall pacing simulations for increasingly long RV-LV delays and with the RV stimulus moved from the RV apex to the RV septum (Figure 3). BIV-endo lateral wall pacing was less sensitive to prolonged RV-LV delays compared to BIV-LBP (Figure 3A, solid lines), and moving the electrode from the RV apex to the RV septum worsened response (Figure 3A, dashed lines). LV lateral wall pacing however remained less sensitive to prolonged RV-LV delay compared to selective LBP. The distribution of simulated activation times in Figure 3B shows that when the RV-LV delay was long (35 ms), BIV-LBP led to similar activation to baseline. In particular, when the RV lead is placed in the septum, LV activation remains unchanged from baseline because when the LV stimulus is fired, the LV septum has already been activated by the RV stimulus, preventing LV septum capture. On the other hand, BIV-endo lateral wall pacing allowed for shorter LV activation. The response metrics in Figure 3C computed for long RV-LV delays (35 ms) show that LVAT-95 during selective LBP and RV septal pacing were similar to baseline (94.8 ± 9.3 ms vs. 94.8 ± 8.3 ms, *p* = 0.9). LVAT-95 and BIVAT-90 were shortened by all other pacing modalities, despite prolonged RV-LV delay. BIV-endo lateral wall pacing attenuated the effect of delayed LV stimulus compared to BIV-LBP (RV apex: BIV-endo lateral: 66.2 ± 7.5 ms vs. LBP: 73.2 ± 8.0 ms, *p* = 0.03; RV septum: BIV-endo lateral: 71.2 ± 7.5 ms vs. LBP: 78.6 ± 7.9 ms, *p* = 0.01). When the RV-LV delay was short, RV septal or apical pacing combined with either selective LBP or LV lateral wall pacing led to similar response. LV lateral wall pacing was however less sensitive to prolonged RV-LV delays compared to LBP.

### The effect of suboptimal left bundle capture

**FIGURE 4:**
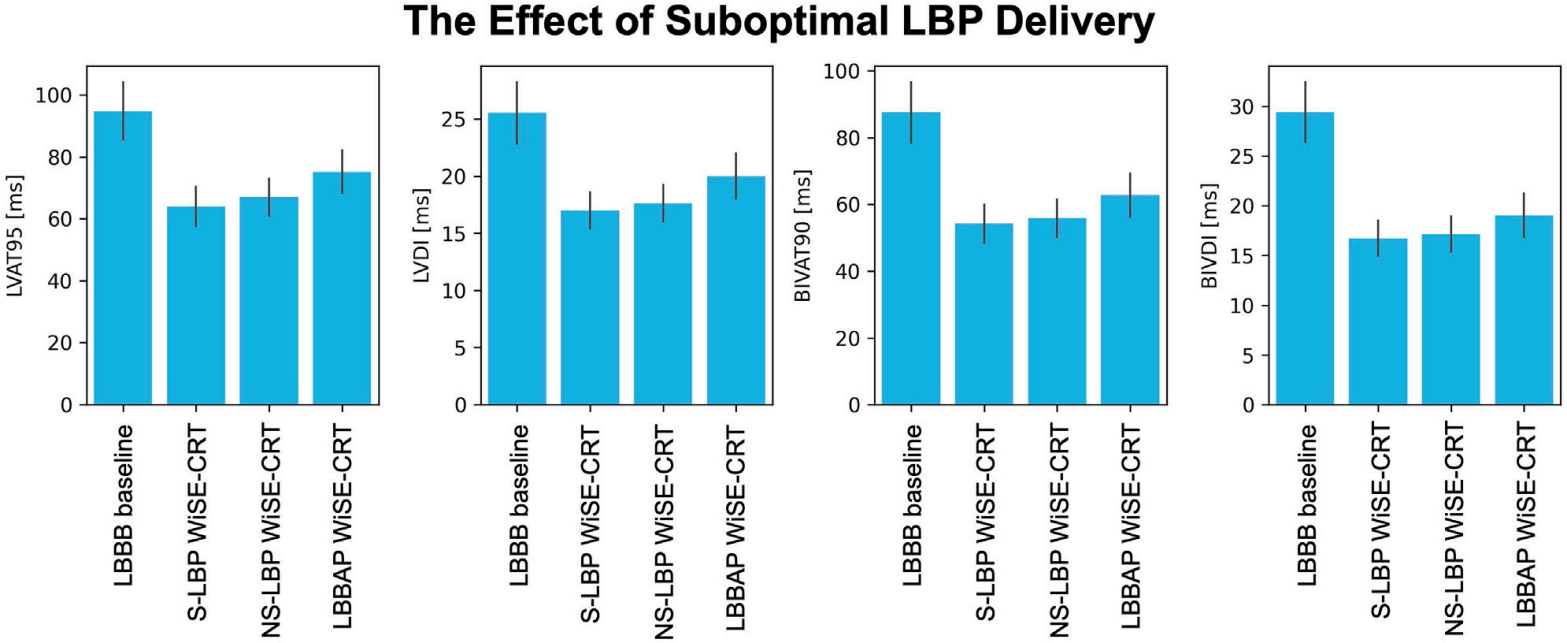
The effect of suboptimal LBP delivery. Response metrics (mean ± standard deviation) simulated during baseline and LBP pacing simulated through the WiSE-CRT system with selective capture, non-selective capture and LBBAP (pacing 1 cm below the left bundle). All pacing simulations were performed with RV apical pacing and 5 ms RV-LV delay.

The simulations presented above assumed perfect selective capture of the left bundle. However, in reality, purely selective LBP is hard to achieve. To test the effect of suboptimal BIV-LBP with a leadless system on response to pacing, we repeated simulations with non-selective LBP and LBBAP, simulated by pacing 1 cm below the left bundle, all combined with an RV apical lead and an RV-LV delay of 5 ms. Figure 4 shows the response metrics simulated during baseline and pacing. Non-selective left bundle capture was comparable to selective capture in terms of LVAT-95 (64.0 ± 6.3 ms vs. 67.0 ± 6.0 ms, *p* = 0.9) and BIVAT-90 (54.2 ± 5.7 ms vs. 55.9 ± 5.6 ms, *p* = 0.9). Although LBBAP significantly worsened response compared to selective LBP (BIVAT-90: 62.7 ± 6.5, *p* < 0.01), ventricular activation was still improved from baseline (*p* < 0.01 for all metrics). Targeting the left bundle selectively or non-selectively does not alter response to BIV-LBP. However, when the left bundle is not targeted correctly, response can worsen significantly.

## Discussion

We carried out an *in silico* clinical trial to investigate response to BIV-endo lateral wall pacing and BIV-LBP delivered through a leadless system in LBBB patients. When AV delay optimization was possible, selective LBP alone through a standard LV lead was more effective than BIV-LBP. However, in the presence of complete AV block, BIV-LBP achieved better synchrony over LBP alone, as the RV pacing stimulus shortened RV activation. We studied the effect of RV-LV delay and RV pacing location on response by increasing the RV-LV delay from 5 ms to 35 ms, and by changing the location of the RV stimulus from apex to septum. BIV-endo lateral wall pacing was less sensitive to prolonged RV-LV delays compared to BIV-LBP, while RV septal pacing worsened response. RV septal pacing combined with LBP with a 35 ms RV-LV delay led to unchanged LV activation from baseline because the LV septum became refractory following RV septal pacing, preventing left bundle capture. To test the effect of the type of left bundle capture on synchrony induced by BIV-LBP, we simulated selective LBP, non-selective LBP and LBBAP. While non-selective LBP was comparable to selective LBP, LBBAP worsened response, although all activation metrics were still significantly improved from baseline.

BIV-endo pacing has emerged as an alternative to conventional CRT for patients who could not receive or did not respond to conventional CRT (Derval et al., 2010; Ginks et al., 2012; Behar et al., 2016; Morgan et al., 2016; Padeletti et al., 2016). However, there are concerns about increased stroke risks, that could be mitigated by performing pacing through a leadless pacing system. The first feasibility study of the WiSE-CRT system reported successful implant in 92% of patients, did not report any thrombo-embolic events. Consistent with our simulation study, these patients achieved a significant enhanced electrical synchrony compared to baseline (Auricchio et al., 2014). Similarly, in the SELECT-LV study 97.1% of patients were successfully delivered with WiSE-CRT system pacing, with significant QRS duration reduction compared to baseline. However, device- or procedure-related complications occurred in 8.6% of patients within the first 24 h, and in 22.9% of patients between 1 day and 1 month, respectively (Reddy et al., 2017). Sieniewicz et al. reported similar complication rates following WiSE-CRT system implantation, improved LV haemodynamics and shortened QRS duration following pacing in the optimal LV endocardial pacing location (Sieniewicz et al., 2020). Despite these promising results, safety of BIV-endo pacing through the WiSE-CRT system needs to be assessed in larger clinical trials, before this technique is widely used (Wijesuriya et al., 2022).

Most studies reporting on the WiSE-CRT system implanted the LV leadless electrode at the LV free wall. However, targeting the left bundle with the LV electrode could potentially provide added benefits thanks to CSP. Elliott et al. reported the first case of leadless LBP (Elliott et al., 2021). The authors tested different locations of the LV lead during BIV-endo pacing, achieving the best acute haemodynamic response by pacing in the LV mid-lateral wall. Consistent with our study, both BIV-endo lateral pacing and BIV-LBP through the WiSE-CRT system significantly improved electrical synchrony compared to baseline. LBP allowed for superior QRS narrowing compared to BIV-endo lateral wall (106 ms vs. 132 ms). BIV-LBP using the WiSE-CRT system was subsequently performed in a series of eight patients (Elliott et al., 2022). These studies showed the technical feasibility of LBP through a leadless system, however the safety and efficacy of this technique, and the importance of targeting of the left bundle, remains unclear. Our results mimic the pacing study that was performed in (Elliott et al., 2022), and showed that, although selective LBP alone is better when AV delay optimization, BIV-LBP is more effective when AV delay optimization is not possible. Although typically the WiSE-CRT system delivers BIV pacing, LV only pacing could be achieved with sub-capture RV pacing output or with further device modification as shown by (Elliott et al., 2022). This could be particularly relevant not only in patients with complete AV block, but also for patients with atrial fibrillation, who represent a significant proportion (about 26%) of CRT patients (Dickstein et al., 2018). On the other hand, patients with RBBB or septal scar are unlikely to respond to leadless LBP due to preserved delayed RV activation during pacing, as we have shown in a previous modelling study (Strocchi et al., 2022) and in the supplement, respectively. In these patient groups, leadless BIV-endo lateral wall pacing might be a better treatment option. Finally, we have shown that longer RV-LV delays worsen response. Studies have reported superiority of optimized RV-LV delay compared to simultaneous BIV pacing, with LV or RV pre-pacing being beneficial for different patients (Sogaard et al., 2002). Other clinical studies instead have reported that RV-LV delay optimization brings no additional benefits to CRT (Boriani et al., 2006; Rao et al., 2007; Bogaard et al., 2013). Therefore, the RV-LV delay is likely to be patient specific and highly dependent on the electrical substrate causing dyssynchrony. While RV-LV delay optimization could be achieved in future with WiSE-CRT through device modifications, it is currently not possible to set a specific RV-LV delay. The conclusions of our study provide insight into response to leadless pacing, and which device parameters are important for response to pacing. This will help in the design of larger clinical trials investigating the efficacy and safety of leadless pacing.

### Limitations

The main limitation of our study is that we assume acute electrical response correlates with long-term functional response. However, although other factors affect response to CRT, there is strong evidence showing that patients who respond acutely to pacing in terms of QRS narrowing are more likely to experience long-term benefits (Bryant et al., 2013; Bazoukis et al., 2020).

The study presented in this paper accounts for a limited number of patients. Even if the twenty-four geometries we used are representative of the heart failure population with CRT indication, a much larger number of meshes should have been considered to model the large heterogeneity observed in patients with dyssynchrony. Over the next decade, the progress in image analysis, segmentation and simulation software will hopefully allow for larger virtual clinical trial including >1000 patients, consistent with large multi-centre trials (Bristow et al., 2004).

Our models make use of synthetic His-Purkinje systems that do not represent the conduction system of a specific patient. However, at present, patient-specific His-Purkinje networks cannot be generated due to the lack of imaging techniques able to resolve these intricate structures. The electrophysiology model we employed was simplified as it discarded cellular ionic dynamics, tissue heterogeneities within the myocardium and electrical signal propagation across the torso. More detailed and personalized simulations would have required more extensive computational resources and were outside the scope of this study. We showed in the supplement that our models replicate baseline metrics and activation pattern of LBBB. However, due to the rule-based His-Purkinje network and the simplified electrophysiology model we employed, the results presented in this study should be interpreted with care.

Despite its limitation, our *in silico* trial succeeds in providing insight into response to pacing delivered through a leadless system, and how RV lead location, LV lead location, RV-LV delay and type of LBP capture alter synchrony. The results we presented lay the foundation for clinical trial design investigating leadless pacing safety and efficacy.

## Conclusion

When AV delay optimization is possible, selective LBP delivered through a standard LV lead (e.g., no RV pacing) offers better synchrony compared to BIV-LBP, while delayed RV activation makes LBP less effective when AV delay optimization is not possible. BIV-endo lateral wall pacing is less sensitive to prolonged RV-LV delays, and RV septal pacing worsens response compared to RV apical pacing, especially for longer RV-LV delays. Non-selective capture of the left bundle is comparable to selective LBP, while LBBAP worsens response compared to selective LBP.

## Data Availability

The raw data supporting the conclusions of this article will be made available by the authors, without undue reservation.
