# Supplementary material for: Leadless biventricular left bundle and endocardial lateral wall pacing versus left bundle only pacing in left bundle branch block patients
Source: Front Physiol. 2022 Dec 14;13:1049214. doi: 10.3389/fphys.2022.1049214 (PMC9794756; doi:10.3389/fphys.2022.1049214)
Supplement: Supplementary file 1 [file DataSheet1.pdf]

Supplementary Material

Supplementary Material

1 His-Purkinje Network Generation

The His-Purkinje network generation method was based on Gillette et al[1] and is described in detail in the original publication. Figure 1 shows the inputs for the His-Purkinje network generation pipeline (top row). The Purkinje tree is grown on the left ventricle (LV) and on the right ventricle (RV) endocardial surfaces (red surfaces in Figure 1, top row), excluding the base, as this was previously shown to lead to more physiological sinus rhythm activation.[2] Universal ventricular coordinates (UVCs) were defined on the ventricles (Figure 1, top row), consisting of an apico-basal coordinate, ranging continuously from 0 to 1 from apex to base, a transmural coordinate, defined as 0 at the endocardium and 1 at the epicardium, and a rotational coordinate, rotating around the ventricles from -π to 0 from the LV free wall to the septum and then back from 0 to +π from the septum back to the LV free wall.

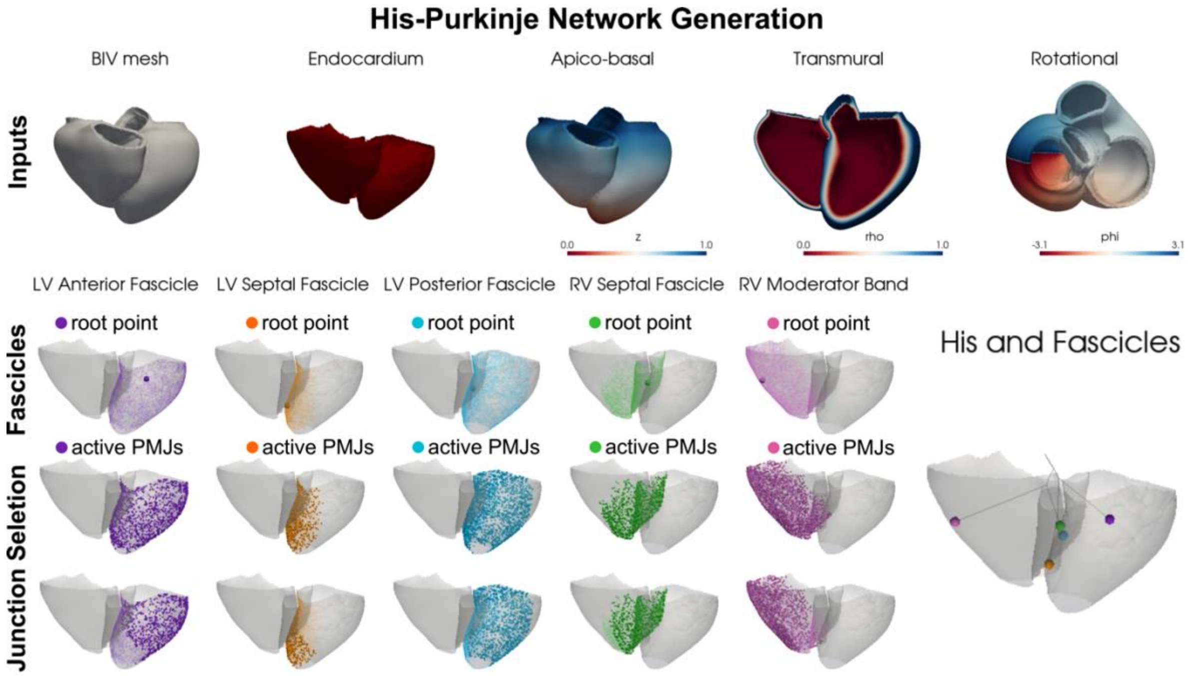

Figure 1 His-Purkinje network generation pipeline. The first row shows the inputs for the His-Purkinje Network generation (from left to right): a biventricular mesh, the left and right ventricular endocardial surfaces the network is grown on, an apico-basal universal ventricular coordinate (UVC), ranging between 0 at the apex and 1 at the base, a transmural UVC, varying from 0 to 1 from the endocardium to the epicardium and a rotational UVC from -π to +π around the ventricles. The second row shows the root points for the five fascicles included in the modes. The third and rows represent all Purkinje-myocardium junctions (PMJs) and the final PMJs, remaining after deactivating the redundant PMJs. On the right, a picture of the His and fascicles, showing the root points as colored spheres.

The model accounts for three LV fascicles (colors refer to Figure 1, second to fourth row): anterior (purple), septal (orange) and posterior (light-blue), and for two RV fascicles: septal (green) and moderator band (pink).

The location of the root points (second row, Figure 1) was provided in terms of UVCs and was based on early activated areas in the Durrer maps. [3] The root points were then used to grow five independent networks that were joined to the His as shown in Figure 1, bottom-right. The His bundle is formed by filaments bundled together and insulated within a common cable.[4] These filaments are predestined to either the left bundle or the right bundle. To represent this anatomical property of the His bundle, we duplicated the His bundle segments in our Purkinje networks. One strand continues into the left bundle and the other continues into the right bundle.

The trees associated with the fascicles are grown independently from each other and can therefore overlap. This would not make a difference in sinus rhythm simulations, where the stimulus spreads from the His, fascicles, Purkinje and finally to the myocardium through the Purkinje-myocardium junctions (PMJs), e.g. the terminal points of the His-Purkinje network, as each node can activate only once. On the other hand, during pacing, additional PMJs can affect the simulation as the stimulus can enter the wrong fascicle. To prevent this, we deactivated redundant PMJs. First, the PMJs associated with each fascicle were found (Figure 1, third row). We then identified the portions of LV and RV endocardial surfaces that were covered by each fascicle tree, and we identified areas where the trees overlapped. An eikonal simulation was run on the tree associated with each fascicle by stimulating the root point to find when each terminal point would activate. In areas where the fascicle trees overlapped, only the PMJs with the shortest activation time were connected with the surrounding myocardium and were therefore activated. The PMJs remaining active after this procedure are shown in Figure 1, bottom row. The image shows that there is no overlap between active PMJs belonging to different fascicles.

The timing of the first activations from the fascicles to ventricular myocardium was based on the Durrer maps.[3] The first LV activation occurs at the three simultaneous fascicle locations (anterior, septal and posterior). Therefore, we computed the conduction velocity (CV) of each LV fascicle separately to guarantee simultaneous activation of the root points of the fascicles. According to the Durrer maps, the RV fascicles activate about 10 ms later than the LV fascicles. Therefore, the CV of the RV fascicles was computed to achieve activation of the RV fascicles root points 10 ms later than the LV.

This network generation pipeline was applied to all twenty-four patient-specific meshes. Proximal LBBB was simulated by disconnecting the left bundle branch from the LV Purkinje network along the His.

2 Model validation

We validated the baseline model using electrocardiographic imaging (ECGi) during left bundle branch block (LBBB) acquired from 8 LBBB patients as part of two ECGi studies.[5,6] Below, we describe the available clinical data and compare simulation results against them.

2.1 Electrocardiographic Imaging data

ECGi data were collected as part of two clinical studies.[5,6] The first study[6] (trial registration number NCT01831518) recruited 11 patients undergoing CRT implantation, including LBBB (N=7) and non-LBBB (N=4) baseline rhythm. For the purpose of this study, we compared our LBBB model against data from 7 LBBB patients. The details about data collection and analysis have been published previously [6]. Briefly, ventricular epicardial activation times were computed from unipolar electrograms as the time of maximum negative slope during the QRS complex. Then, we computed metrics to quantify left ventricular (LV) and biventricular (BIV) activations, similar to what we did for the simulation results: shortest time interval needed to activate 95% of the LV (LVAT95); LV dyssynchronous index (LVDI), computed as the standard deviation of LV activation times; shortest time interval needed to activate 90% of the ventricles (BIVAT90), BIV dyssynchronous index (BIVDI), computed as the standard deviation of ventricular activation times; ventricular electrical uncoupling (VEU) [7], computed as the difference between mean LV activation times and mean right ventricular (RV) activation times. The metrics computed from ECGi data accounted only for the ventricular epicardial surface, while the metrics computed from simulation results accounted for the whole ventricular myocardium. In both cases, the base of the ventricles, including the outflow tracts, was excluded from the mesh. We analyzed 3 baseline beats for each patient, leading to a total of 21 beats. We considered one additional LBBB patient from a second study[5] (trial registration number NCT 04322877) who also underwent ECGi. 3 baseline LBBB beats were collected and analyzed.

2.2 LBBB Baseline Validation

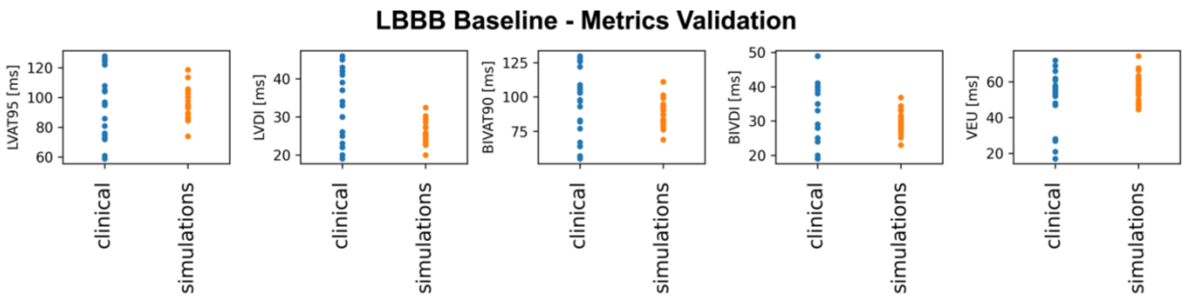

Figure 2 Comparison between ECGi metrics (N=24 beats) from LBBB patients at baseline in blue and metrics predicted by the model (N=24 patients) during LBBB baseline in orange. From left to right: LVAT95, LVDI, BIVAT90, BIVDI and VEU.

Figure 2 shows the comparison between the LBBB baseline metrics measured from the 24 beats extracted from the ECGi data and the metrics predicted by the model for all 24 patients. All metrics are within physiological ranges, showing that the model can reproduce features of LBBB activation pattern. We also show that the model replicates local LBBB activation by comparing an anterior and a posterior view of the epicardial maps for one patient at baseline with the epicardial activation times predicted by the model for all patients (Figure 3 and 4).

The model validation provided in this section shows that the baseline LBBB model reproduces standard LBBB activation patterns with large delays between the RV and the LV activations. We also show that metrics extracted from the model agree with the metrics computed from ECGi data. Therefore, we can conclude that the model can be used to make considerations about response to pacing.

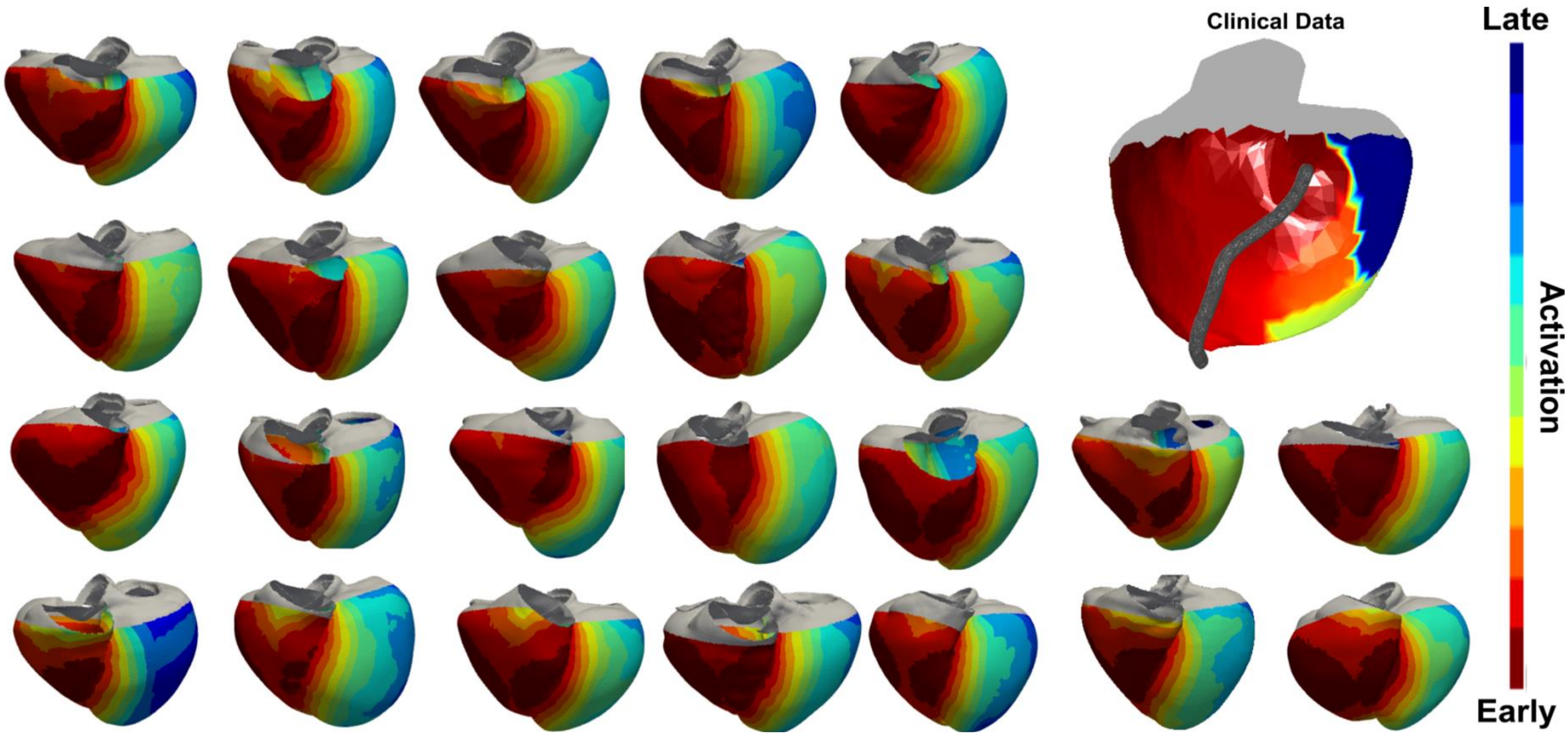

Figure 3 Anterior view of the comparison between epicardial activation measured from ECGi data for one patient (top right) and epicardial activation times predicted by the model for all twenty-four geometries. Red to blue areas show early to late activated regions, while gray areas represent the base of the ventricles, excluded from metrics computation.

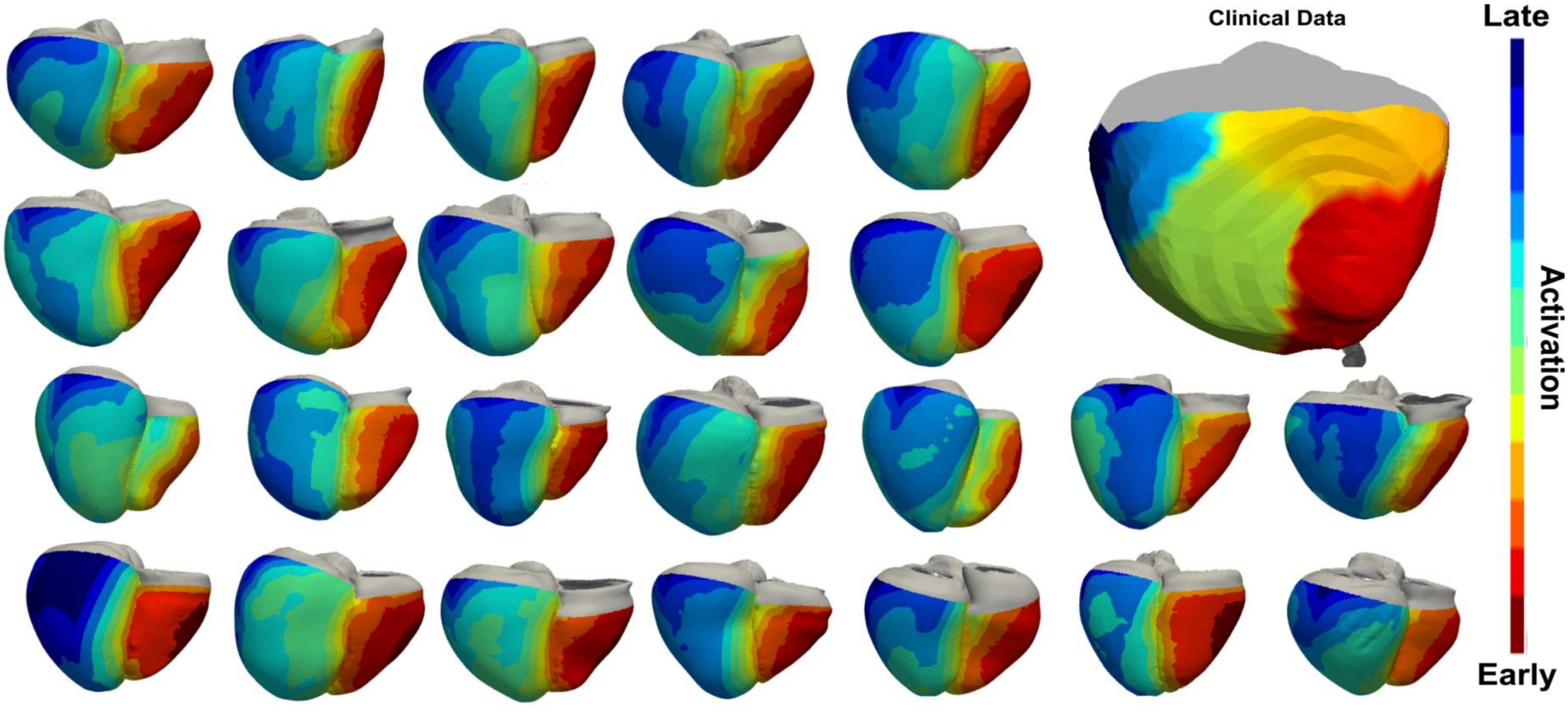

Figure 4 Posterior view of the comparison between epicardial activation measured from ECGi data for one patient (top right) and epicardial activation times predicted by the model for all twenty-four geometries. Red to blue areas show early to late activated regions, while gray areas represent the base of the ventricles, excluded from metrics computation.

3 The effect of septal scar on simulation results

We mapped a patient-specific septal scar and border zone geometries onto all twenty-four meshes to simulate the effect of septal scar on the results presented in the manuscript. We simulated baseline LBBB, selective LBP with and without optimized atrioventricular (AV) delay, leadless left bundle pacing (BIV-LBP) and leadless pacing with the LV lead placed in the free wall. The activation metrics were then computed as described in the methods of the manuscript and compared.

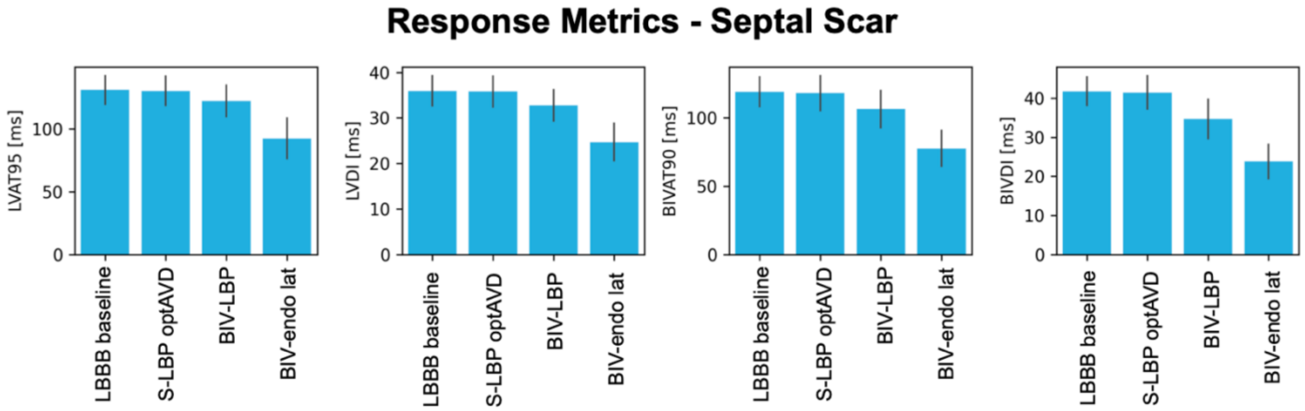

Figure 5 LVAT-95, LVDI, BIVAT-90 and BIVDI are shown for baseline, selective LBP with optimized AV delay (S-LBP optAVD), leadless pacing with the LV lead targeting the left bundle (BIV-LBP) and in the lateral wall (BIV-endo lat). The bar plot represents the mean while the black segments represent ± standard deviation.

Figure 5 shows the left ventricular 95% activation time (LVAT95), the left ventricular dyssynchronous index (LVDI), the biventricular 90% activation time (BIVAT90) and the biventricular dyssynchronous index (BIVDI) simulated during baseline and pacing in the presence of septal scar. Results for selective LBP without optimized AV delay are not reported because the stimulus did not result in any activation due to the present of non-conducting scar surrounding the stimulus region. Selective LBP with optimized AV delay resulted in the same activation metrics as baseline as the LBP stimulus does not capture due to the presence of scar (LVAT95: baseline vs pacing 130.9±11.2 ms vs 130.2±11.6 ms, P=0.9; BIVAT90: baseline vs pacing 119.1±10.8 ms vs 118.2±12.6 ms, P=0.9). Compared to baseline, leadless pacing with the leadless electrode targeting the left bundle (BIV-LBP) slightly improves BIV (BIVAT90: 119.1±10.8 ms vs 106.4±13.6 ms, P<0.01) but not LV activation times (LVAT95: 130.9±11.2 ms vs 122.2±12.5 ms, P=0.1). This is due to the LBP stimulus not capturing, with changes in activation due only to right ventricular pacing. Leadless pacing with the LV electrode placed in the LV free wall is the most effective, as it reduces both LV (LVAT95: 130.9±11.2 ms vs 92.3±16.1 ms, P<0.01) and BIV (BIVAT90: 119.1±10.8 ms vs 77.6±13.0 ms, P<0.01) activation compared to baseline. This is because the septal scar is away from the LV stimulus site, increasing pacing efficacy.

LBP performed with either a lead-based or a leadless system is ineffective in patients with septal scar, as the LBP stimulus does not capture healthy myocardium. On the other hand, BIV-endo lateral wall pacing efficacy is not affected by the presence of septal scar.

4 References

1. Gillette K, Gsell MAF, Bouyssier J, et al. Automated Framework for the Inclusion of a His–Purkinje System in Cardiac Digital Twins of Ventricular Electrophysiology. *Ann Biomed Eng*. 2021;49(12). doi:10.1007/s10439-021-02825-9

2. Strocchi M, Lee AWC, Neic A, et al. His-bundle and left bundle pacing with optimized atrioventricular delay achieve superior electrical synchrony over endocardial and epicardial pacing in left bundle branch block patients. *Heart Rhythm*. 2020;17(11). doi:10.1016/j.hrthm.2020.06.028

3. Durrer D, van Dam RT, Freud GE, Janse MJ, Meijler FL, Arzbaecher RC. Total excitation of the isolated human heart. *Circulation*. 1970;41(6). doi:10.1161/01.CIR.41.6.899

4. Vijayaraman P, Chung MK, Dandamudi G, et al. His Bundle Pacing. *J Am Coll Cardiol*. 2018;72(8):927-947. doi:10.1016/j.jacc.2018.06.017

5. Elliott MK, Mehta V, Sidhu BS, Niederer S, Rinaldi CA. Electrocardiographic imaging of His bundle, left bundle branch, epicardial, and endocardial left ventricular pacing to achieve cardiac resynchronization therapy. *HeartRhythm Case Rep*. 2020;6(7). doi:10.1016/j.hrcr.2020.04.012

6. Elliott MK, Strocchi M, Mehta VS, et al. Dispersion of repolarization increases with cardiac resynchronization therapy and is associated with left ventricular reverse remodeling. *J Electrocardiol*. Published online 2022.

7. Ploux S, Eschalier R, Whinnett ZI, et al. Electrical dyssynchrony induced by biventricular pacing: Implications for patient selection and therapy improvement. *Heart Rhythm*. 2015;12(4). doi:10.1016/j.hrthm.2014.12.031
